# Future perspectives: targeting fibroblast growth factor receptor 1 to enhance the efficacy of immunotherapy

**DOI:** 10.37349/etat.2025.1002327

**Published:** 2025-06-20

**Authors:** Ilya Tsimafeyeu

**Affiliations:** IRCCS Istituto Romagnolo per lo Studio dei Tumori (IRST) “Dino Amadori”, Italy; Bureau for Cancer Research - BUCARE, New York, NY 10032, USA

**Keywords:** Fibroblast growth factor receptor 1, anti-FGFR1 antibody, immune checkpoint inhibitors, resistance

## Abstract

Fibroblast growth factor receptor 1 (FGFR1) plays a critical role in the progression of various cancers through its involvement in cell proliferation, survival, and differentiation. More recently, FGFR1 has been implicated in the mechanisms of immune evasion, particularly its role in resistance to immune checkpoint inhibitors (ICIs) such as pembrolizumab and nivolumab. Targeting FGFR1 with monoclonal antibodies and tyrosine kinase inhibitors has emerged as a promising therapeutic strategy to enhance ICI efficacy by altering the tumor microenvironment and countering immune suppression. Preclinical studies demonstrate that combining FGFR1 inhibitors, such as the novel monoclonal antibody OM-RCA-01, with ICIs significantly improves antitumor activity, enhancing T cell responses and cytokine production. This article explores the role of FGFR1 in cancer biology, its contribution to immunotherapy resistance, and the therapeutic potential of targeting FGFR1 to enhance the efficacy of ICIs.

## Introduction

The introduction of immune checkpoint inhibitors (ICIs) has revolutionized cancer treatment, particularly for tumors that evade the immune system by expressing proteins such as programmed death-ligand 1 (PD-L1) and inhibiting T cell activity [1]. However, a significant subset of patients does not respond to ICIs due to intrinsic resistance mechanisms including the tumor microenvironment (TME) [2, 3].

Among the various molecular players in cancer progression, fibroblast growth factor receptor 1 (FGFR1) has emerged as a critical target for therapeutic intervention [4]. FGFR1 belongs to the FGFR family of receptor tyrosine kinases, which are involved in numerous biological processes. Dysregulation of FGFR1 has been observed in several cancers, including lung cancer, breast cancer, renal cell carcinoma (RCC), and head and neck tumors, where its overexpression is often associated with poor prognosis [5–8]. Furthermore, FGFR1 has been implicated in promoting resistance to ICIs by modulating the immune microenvironment and enabling immune evasion [9].

Given the pivotal role of FGFR1 in tumor biology and immune evasion, targeting FGFR1 represents a promising approach to enhance the efficacy of immunotherapy. This perspective will explore the biological significance of FGFR1 in cancer progression, the mechanisms by which it contributes to immunotherapy resistance, and the emerging evidence supporting FGFR1-targeting therapies in combination with ICIs.

## FGFR1-mediated mechanisms in cancer progression and immunotherapy resistance

### Role of FGFR1 in tumor growth and progression

**Figure 1:**
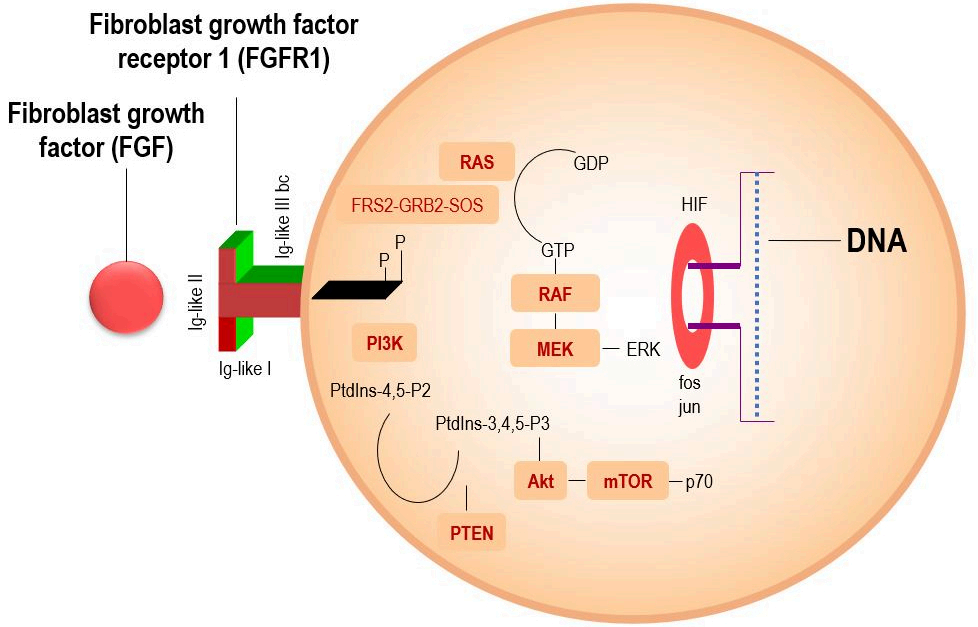
**A scientific diagram illustrating the mechanism of FGFR1 signaling, showing key pathways like PI3K-Akt-mTOR and RAS-RAF-MEK-MAPK for cell survival and proliferation.** Adapted with permission from [https://sciencefeatured.com/2024/11/28/a-drug-candidate-an-anti-fgfr1-humanized-antibody-offers-fresh-approach-to-battling-aggressive-lung-cancer/], cited 2025 May 10, © 2021 Science Featured is an entity of Science Bridges, a non-profit Canadian corporation

In normal tissues, FGFR1 plays an essential role in embryonic development and tissue homeostasis [10]. However, in cancer, dysregulation of FGFR1 signaling, often due to gene amplification or overexpression, leads to uncontrolled cell proliferation and tumorigenesis [11, 12]. FGFR1 is one of the four members of the FGFR family, which includes FGFR1, FGFR2, FGFR3, and FGFR4. Activation of FGFR1 by its ligands, fibroblast growth factors (FGFs), leads to the phosphorylation of the tyrosine kinase and downstream signaling through the Ras-MAPK, PI3K-Akt, and JAK/STAT pathways (Figure 1) [10]. These signaling cascades promote cell survival, proliferation, and angiogenesis, contributing to tumor growth. FGFR1 amplification or overexpression has been identified in a variety of cancers, where it drives oncogenic processes. In non-small cell lung cancer (NSCLC), for example, FGFR1 amplification has been reported in approximately 20% of cases, and its overexpression of up to 13% is associated with a more aggressive tumor phenotype [5, 13].

FGFR1 involvement in angiogenesis is also significant, as it further contributes to tumor proliferation and the spread of metastases [4]. Cross-activation of FGFR with other cell surface proteins has also been described [14, 15].

Numerous sequencing studies have revealed a wide range of FGFR1 abnormalities across tumor patients, with prevalence rates spanning 1.9% to 98% [16]. Among these abnormalities, gene amplifications are the most frequent (53.7–66%), followed by mutations (26–38.8%) and rearrangements or fusions (5.6–8%). Table 1 summarizes the prevalence and notable FGFR1 abnormalities in various tumor types.

**Table 1:** Prevalence of FGFR1 abnormalities in various tumor types

**Tumor type**	**Amplifications (%)**	**Mutations (%)**	**Gene rearrangements (%)**	**Overexpression (%)**
Lung cancer (squamous cell carcinoma)	10–56.8	N546K, K656E, V561M (varied)	FGFR1-TACC1 (not quantified)	4.4–13
Breast cancer	7–15	S125L, K566R (0.19)	NR	6.1–58
Glioma/glioblastoma	2–66.7	N546K, K656E (3–21)	FGFR1-TACC1 (2–4)	Rare
Prostate cancer	NR	NR	NR	20–40
Head and neck tumors	6–17	NR	NR	10.6–82
Renal cell carcinoma	NR	NR	NR	98 (primary), 82.5 (metastatic)
Myeloproliferative disorders	NR	NR	BCR-FGFR1, FGFR1OP-RET, FGFR1OP-FGFR1 (common)	Rare

NR: not reported; FGFR1: fibroblast growth factor receptor 1

FGFR1 aberrations occur at frequencies of 49–56.8%, with FGFR1 amplification linked to unfavorable clinical outcomes [16]. For instance, patients with squamous cell lung cancer and FGFR1 amplification demonstrate significantly reduced overall survival (OS) compared to those without amplification (58.6 months vs. 80.0 months) [17]. FGFR1 amplification also appears in approximately 10% of breast cancers, driving increased ligand-dependent signaling, suppressing progesterone receptor expression, and correlating with poor prognosis [18].

FGFR1 mutations, such as N546K, K656E, and V561M, typically occur within the kinase domain, leading to aberrant receptor activation and persistent pathway signaling [16, 19]. These mutations are implicated in various malignancies, including H3K27M-mutant diffuse midline gliomas (DMGs), where FGFR1 point mutations (N546K and K656E) are found in 21% of cases. This subset is more prevalent among older individuals with diencephalic tumors and is associated with increased malignancy, reduced sensitivity to FGFR inhibitors, and spontaneous hemorrhage. Similar findings have been reported in other central nervous system cancers, further highlighting their role in aggressive tumor behavior.

Gene rearrangements, involving the rearrangement of genes on chromosomes, also impact FGFR1 function. FGFR1 fusions with various partner genes have been identified in several tumor types [16]. For example, the FGFR1-TACC1 fusion in glioblastoma and squamous cell lung cancer leads to FGFR1 hyperactivation, enhancing cell proliferation and inhibiting apoptosis. This fusion may increase FGFR1 tyrosine kinase activity or alter its intracellular localization. Other notable fusions, such as BCR-FGFR1, FGFR1OP-RET, and FGFR1OP-FGFR1, are associated with myeloproliferative disorders, contributing to disease onset and progression by promoting cell cycle progression and suppressing apoptosis.

Finally, FGFR1 overexpression is also critical in tumor pathogenesis [5, 7]. For example, in RCC, FGFR1 expression is detected in 98% of primary tumor cells and 82.5% of metastatic cells in lymph nodes [4]. Expression rates in other tumor types vary widely, including lung cancer (4.4–13%) [5, 20], breast cancer (6.1–58%) [21, 22], prostate cancer (20–40%) [23, 24] and head and neck tumors (10.6–82%) [25]. In all these cancers, FGFR1 plays a significant role in driving tumor development and progression.

### FGFR1 and immunotherapy resistance

**Figure 2:**
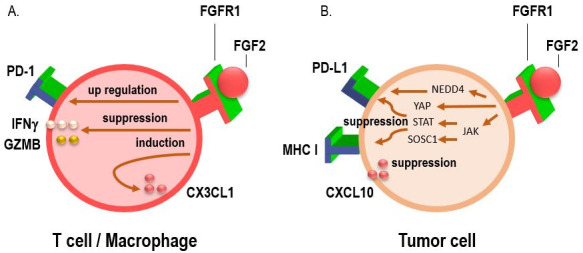
**Interactions between the PD-1/programmed death-ligand 1 (PD-L1), fibroblast growth factor receptor 1 (FGFR1) pathways, and chemokines.** (**A**) Activation of the FGF/FGFR signaling pathway has been shown to upregulate PD-1 expression on effector T cells, while simultaneously suppressing the secretion of key cytotoxic mediators such as interferon-gamma (IFNγ) and granzyme B (GZMB). This dual effect contributes to diminished T cell-mediated antitumor immune responses, highlighting a potential mechanism of immune evasion in FGFR-driven tumors. In addition, FGFR1 activation facilitates macrophage recruitment to the tumor microenvironment by inducing the expression of the chemokine CX3CL1, thereby contributing to an immunosuppressive milieu that supports tumor progression; (**B**) FGF/FGFR signaling has been shown to suppress IFN-induced immune activation by downregulating the expression of MHC class I molecules, PD-L1, and the chemokine CXCL10. This immunosuppressive effect is mediated, in part, through the induction of suppressor of cytokine signaling 1 (SOCS1), which interferes with downstream IFN signaling. Conversely, FGF/FGFR signaling can also enhance PD-L1 expression via alternative pro-tumorigenic pathways. Specifically, activation of the JAK/STAT signaling cascade leads to transcriptional upregulation of PD-L1, while concurrent stimulation of the Hippo pathway effector YAP further promotes PD-L1 transcription. Together, these mechanisms contribute to tumor immune evasion by inhibiting antigen presentation and suppressing effector T cell recruitment and function. Conversely, FGFR1 signaling has also been implicated in the post-translational regulation of PD-L1 stability. Specifically, FGFR1 promotes the phosphorylation of the E3 ubiquitin ligase NEDD4, which in turn facilitates the ubiquitin-mediated proteasomal degradation of PD-L1. This mechanism may act as a counterbalance to transcriptional upregulation pathways, potentially modulating PD-L1 expression levels in a context-dependent manner and influencing tumor immune escape dynamics

Several studies have investigated the effects of FGFR on tumor immunity. FGFR1 has been shown to modify the TME in a manner that favors immune evasion, particularly by upregulating immune checkpoint molecules such as PD-L1, which inhibits cytotoxic T cell function and allows tumors to escape immune surveillance (Figure 2) [15, 26, 27]. Additionally, FGFR1 signaling can recruit immunosuppressive cells, such as regulatory T cells (Tregs) and myeloid-derived suppressor cells (MDSCs), further promoting an immune-permissive environment that protects the tumor from immune attack [28, 29]. FGFR1 signaling may also promote the secretion of immunosuppressive cytokines like transforming growth factor-beta (TGF-β), which further dampens the immune response [30–32]. FGFR1 is a component of the microenvironment in bone metastases, enhances osteoclast activity and cytokine release as well as contributes to the formation of metastatic lesions [33–35]. These interactions between FGFR1 and the immune system highlight the need for novel therapeutic strategies that can target FGFR1 while simultaneously enhancing the efficacy of immunotherapy.

The TME can be altered to reduce immunosuppression, potentially reversing resistance to ICIs. Finally, we have previously shown that FGFR expression occurs in human lymphocytes [12]. Therefore, it cannot be ruled out that targeting FGER1 affects the antitumor effect of cytotoxic T cells. This opens avenues for combination therapies where FGFR1-targeted treatments with ICIs [36].

## Targeting FGFR1: a promising therapeutic strategy

FGFR1 has been explored as a therapeutic target in oncology for over a decade. Multiple strategies have emerged to block FGFR1 activity, including the use of tyrosine kinase inhibitors (TKIs) and monoclonal antibodies (Table 2). Various TKIs targeting FGFR1 have undergone clinical evaluation in patients with FGFR1-amplified malignancies. Despite some promise, these agents often encounter hurdles such as acquired resistance and unintended interactions that undermine their long-term effectiveness. Examples of broad-spectrum FGFR inhibitors include BGJ398, AZD4547, and JNJ-42756493, all of which act on FGFR1–3 [37–39]. Though preclinical outcomes were encouraging, translation into clinical benefit has been limited. For instance, in a phase I study, only 11% of FGFR1-amplified NSCLC patients exhibited partial responses to BGJ398.

**Table 2:** Potential therapeutic strategy of FGFR1 inhibition

**Strategy**	**Agent(s)**	**Target/Mechanism**	**Key outcomes**	**Limitations/Notes**
Non-selective FGFR TKIs	BGJ398, AZD4547, JNJ-42756493	FGFR1–3 inhibition (intracellular TK domain)	Limited efficacy; e.g., 11% PR in FGFR1-amplified NSCLC (BGJ398)	Resistance, off-target toxicity, limited durable responses
Selective FGFR TKIs	Pemigatinib	FGFR1 rearrangement (hematologic malignancies)	78% CR in relapsed/refractory MLNs (FIGHT-203 study); FDA approved	High efficacy in specific FGFR1-fusion-driven hematologic cancers
	Alofanib	Selective extracellular FGFR inhibitor	Promising preclinical and clinical data	FGFR2-specific
	Futibatinib	Pan-FGFR inhibitor	Ongoing trials in FGFR1-positive tumors	Still under clinical investigation
Monoclonal antibodies	OM-RCA-01	Binds FGFR1 extracellular domain	Decrease proliferation in lung/RCC cells; tumor growth inhibition in vivo; decrease FGFR1 phosphorylation, high specificity	Ineffective in low-FGFR1 phosphorylation models (e.g., melanoma); still under clinical investigation
Combination with ICIs	OM-RCA-01 + nivolumab	FGFR1 inhibition + PD-1 blockade	Increase IFNγ (33%), increase IL-2 (74%); synergistic tumor suppression in FGFR1+/PD-L1+ lung cancer model	Lack of monotherapy control arm; mechanism of synergy not fully understood
CAF-targeted immunotherapy combination	OM-RCA-01 + nivolumab in CAF-positive TME	Immune evasion reversal via FGFR1 inhibition	Decrease tumor growth; restored IFNγ/IL-2 secretion; mitigated CAF-driven resistance	Further mechanistic exploration needed

FGFR: fibroblast growth factor receptor; TKIs: tyrosine kinase inhibitors; PR: partial response; NSCLC: non-small cell lung cancer; CR: complete response; MLNs: myeloid/lymphoid neoplasms; RCC: renal cell carcinoma; ICIs: immune checkpoint inhibitors; IFNγ: interferon-gamma; PD-L1: programmed death-ligand 1; CAF: cancer-associated fibroblast; TME: tumor microenvironment

Conversely, treatments have shown greater promise in relapsed or refractory myeloid/lymphoid neoplasms (MLNs) with FGFR1 rearrangement. The efficacy of pemigatinib was assessed in the multicenter FIGHT-203 trial, which included 28 such patients [40], with 78% achieving durable complete responses—supporting subsequent FDA approval [41]. Another approach gaining traction is the development of more selective FGFR inhibitors. Alofanib, targeting FGFR2 specifically, has demonstrated strong activity in both preclinical and early-phase clinical studies [42, 43]. Similarly, futibatinib, a selective pan-FGFR blocker, is being studied in FGFR1-positive cohorts [44].

Monoclonal antibodies that bind the extracellular portion of FGFR1 represent a more targeted modality. One example, OM-RCA-01, is a humanized antibody designed to inhibit FGFR1 activation and downstream signaling cascades. In our investigation, we evaluated the capacity of OM-RCA-01 to suppress tumor cell proliferation in vitro and impede tumor progression in vivo. We further hypothesized that combining this antibody with ICIs could improve treatment responses, particularly by addressing resistance mechanisms tied to the TME.

In vitro data showed that OM-RCA-01 significantly diminished FGF-driven proliferation in lung and renal carcinoma cell lines [45, 46], accompanied by a dose-dependent reduction in FGFR1 phosphorylation. In melanoma cells, where baseline FGFR1 phosphorylation was low, the antibody exhibited limited antiproliferative effects. Other studies have similarly linked FGFR1 phosphorylation to cellular proliferation [47–49], reinforcing the notion that extracellular receptor blockade impairs intracellular kinase signaling. Future work should examine downstream kinase phosphorylation in response to OM-RCA-01. Prior findings from other FGFR-targeted agents indicate that extracellular domain inhibition can also disrupt intracellular signaling proteins [50, 51].

These findings were corroborated in vivo using a lung cancer xenograft model [45]. Mice treated with non-specific IgG developed rapidly growing tumors that reached approximately 2,000 mm^3^, whereas OM-RCA-01 monotherapy substantially curtailed tumor expansion—achieving a twofold size reduction by day 31. This likely resulted from both direct tumor suppression and reduced angiogenesis. Supporting this, OM-RCA-01 previously outperformed bevacizumab in a Matrigel-based angiogenesis assay [46].

The next phase of our study focused on exploring the potential of combining immunotherapy with anti-FGFR1 targeting. Immune checkpoint blockade has revolutionized cancer treatment, yet resistance remains a significant clinical obstacle. For instance, only about 30% of metastatic lung cancer patients and 48% of kidney cancer patients reach a five-year survival benchmark following checkpoint inhibitor combinations such as pembrolizumab or nivolumab with ipilimumab [52, 53].

This emphasizes the need for novel strategies to enhance immunotherapy durability and counteract resistance. While early-phase investigations have tested FGFR TKIs alongside PD-1 inhibitors [54, 55], no completed studies have evaluated monoclonal FGFR1 antibody combinations. This represents a promising area for further exploration. One current trial, FORTITUDE-102, is assessing the combination of anti-FGFR2 antibody bemarituzumab with nivolumab and chemotherapy in metastatic gastric cancer patients [56].

Our in vitro experiments confirmed that nivolumab augments T cell activation, consistent with prior reports showing elevated interferon-gamma (IFNγ) and IL-2 levels in co-cultures treated with nivolumab or nivolumab/ipilimumab [57, 58]. Notably, adding OM-RCA-01 further increased IFNγ release by 33% and IL-2 by 74%. The underlying mechanisms are still unclear. Previous work from our lab found FGFR expression on human lymphocytes [12], implying a possible direct immunomodulatory effect. Although a nonspecific immunoglobulin response cannot be ruled out, OM-RCA-01 alone did not elicit significant cytokine release, and preclinical toxicology studies did not identify any notable immune stimulation.

In vivo, the OM-RCA-01 plus checkpoint inhibitor combination enhanced therapeutic efficacy in a patient-derived xenograft (PDX) lung cancer model overexpressing FGFR1. While pembrolizumab monotherapy is standard for metastatic NSCLC with ≥ 50% PD-L1 expression, the interplay between PD-L1 and FGFR1 co-expression remains underexplored. A phase 2 study (NIVOFGFR2) in gastric cancer showed diminished nivolumab efficacy when tumors co-expressed PD-L1 and FGFR2 [59]. Our PDX model featured high levels of both FGFR1 and PD-L1, and tumor growth in the control group was aggressive. By contrast, dual treatment with OM-RCA-01 and pembrolizumab achieved a twofold tumor volume reduction compared to checkpoint inhibitor monotherapy. The absence of a treatment arm using OM-RCA-01 alone is a limitation, but comparative results suggest that its therapeutic impact is magnified when combined with pembrolizumab.

As discussed earlier, TME is pivotal in ICI resistance, with cancer-associated fibroblasts (CAFs) playing a central role [60]. CAFs support metastasis through extracellular matrix remodeling, growth factor production, and modulation of angiogenesis, tumor rigidity, and drug response [61]. Various approaches are under development to neutralize CAF-mediated resistance, including altering their composition or activity. In our model, tumor progression accelerated in the presence of CAFs, with nivolumab efficacy markedly reduced [45]. However, the introduction of OM-RCA-01 upon onset of resistance delayed tumor expansion. Tumors in the CAF-positive cohort did not exceed 2,000 mm^3^, in contrast to controls treated with non-specific IgG. Furthermore, cytokine levels such as IFNγ and IL-2 surged again with OM-RCA-01 therapy. Although these data are not sufficient to draw definitive conclusions about OM-RCA-01’s role in modulating the TME, the results suggest potential direct and indirect antitumor effects. Importantly, OM-RCA-01 was well tolerated, showing no adverse events at or above therapeutic dosages. Pharmacokinetic profiles indicated prolonged clearance and sustained plasma levels, pointing to favorable bioavailability and drug persistence.

## Challenges and future directions

Despite the first promising data on FGFR1-targeted therapies, several challenges must be addressed before these therapies can be fully integrated into clinical practice. One of the primary challenges is the development of resistance to FGFR1 inhibitors. Similar to other targeted therapies, cancer cells may develop mutations in the FGFR1 pathway or activate alternative signaling pathways to bypass FGFR1 inhibition, ultimately leading to therapeutic resistance. Understanding these mechanisms is critical for developing strategies to sustain long-term efficacy.

Another challenge lies in the complexity of the TME. While FGFR1 inhibition can reduce immune suppression and enhance ICI efficacy, the TME remains a dynamic and heterogeneous environment. Other immunosuppressive factors, such as Tregs, MDSCs, and immunosuppressive cytokines like TGF-β, may still limit the effectiveness of combination therapies. Therefore, therapeutic strategies that target not only FGFR1 but also additional components of the TME may be necessary to achieve optimal outcomes.

To overcome these challenges, several emerging strategies are being explored. One approach involves developing next-generation FGFR inhibitors with enhanced specificity and reduced toxicity. These novel agents aim to minimize off-target effects and mitigate resistance mechanisms, making them better suited for long-term combination with ICIs. Another area of active research is the identification of predictive biomarkers to guide patient selection as FGFR1 amplification, FGFR1 mutations, and FGFR1 expression levels are being studied to help identify patients most likely to benefit from FGFR1-targeted therapies as single agents or combined with ICIs. The development of reliable biomarkers will be critical for personalizing treatment approaches and improving clinical outcomes. Furthermore, researchers are exploring the use of circulating tumor DNA and other liquid biopsy techniques to dynamically monitor FGFR1 mutations and treatment response over time.

An emerging strategy could be exploring the efficacy of chimeric antigen receptor (CAR) T cell therapy targeting FGFR1. Such approach could further enhance antitumor efficacy by targeting FGFR1-overexpressing tumor cells [62].

Notably, FGFR TKIs augmented the antitumor effect of FGFR1-reactive T cells against human head and neck cancers [63]. These results suggest that FGFR TKIs are potential immune adjuvants for T cell-based immunotherapy. Combination therapy with TKIs and cancer vaccines or ICI could be a novel and potent immunotherapeutic approach to treat aggressive cancers with FGFR expression.

Finally, a new concept is to use the FGFR as a substrate for the attachment of the antibody-drug conjugate to the cancer cell. So far, attempts to create such conjugates have not been successful due to poor tolerability, however, further developments are underway [64, 65].

Large-scale clinical trials are needed to confirm the efficacy and safety of FGFR1-targeted therapies alone or in combination with ICIs. While early-phase trials have shown promising results, larger, randomized studies are required to validate these findings across different cancer types and patient populations. These trials should also explore variations in treatment protocols, such as different dosing regimens and the sequencing of FGFR1 inhibitors and ICIs. The results of these studies will provide crucial insights into how best to implement FGFR1-targeted therapies in clinical practice.

## Abbreviations

CAF: cancer-associated fibroblast
FGFR1: fibroblast growth factor receptor 1
ICIs: immune checkpoint inhibitors
IFNγ: interferon-gamma
NSCLC: non-small cell lung cancer
PD-L1: programmed death-ligand 1
TKIs: tyrosine kinase inhibitors
TME: tumor microenvironment

## Declarations

### Copyright

© The Author(s) 2025.
